# Rewarding Effects of Operant Dry-Licking Behavior on Neuronal Firing in the Nucleus Accumbens Core

**DOI:** 10.3389/fphar.2017.00536

**Published:** 2017-08-15

**Authors:** Enrico Patrono, Jumpei Matsumoto, Hiroshi Nishimaru, Yusaku Takamura, Ikhruud C. Chinzorig, Taketoshi Ono, Hisao Nishijo

**Affiliations:** System Emotional Science, Graduate School of Medicine and Pharmaceutical Sciences, University of Toyama Toyama, Japan

**Keywords:** intra-gastric self-administration, glucose, operant dry-licking, nucleus accumbens, single unit recording

## Abstract

Certain eating behaviors are characterized by a trend of elevated food consumption. However, neural mechanisms mediating the motivation for food consumption are not fully understood. Food impacts the brain-rewarding-system via both oral-sensory and post-ingestive information. Recent studies have reported an important role of visceral gut information in mediating dopamine (DA) release in the brain rewarding system. This is independent of oral sensation, suggesting a role of the gut-brain-DA-axis in feeding behavior. In this study, we investigated the effects of intra-gastric (IG) self-administration of glucose on neuronal firings in the nucleus accumbens (NA) of water-deprived rats. Rats were trained in an operant-licking paradigm. During training, when the light was on for 2 min (light-period), rats were required to lick a spout to acquire the water oral-intake learning, and either an IG self-infusion of 0.4 M glucose (GLU group) or water (H_2_O group). Rats rested in the dark-period (3 min) following the light-period. Four cycles of the operant-licking paradigm consisting of the light–dark periods were performed per day, for 4 consecutive days. In the test session, the same rats licked the same spout to acquire the IG self-administration of the corresponding solutions, without oral water ingestion (dry licking). Behavioral results indicated IG self-administration of glucose elicits more dry-licking behavior than that of water. Neurophysiological results indicated in the dark period, coefficient of variance (CV) measuring the inter-spike interval variability of putative medial spiny neurons (pMSNs) in the NA was reduced in the H_2_O group compared to the GLU group, while there was no significant difference in physical behaviors in the dark period between the two groups. Since previous studies reported that DA release increases CV of MSNs, the present results suggest that greater CV of pMSNs in the GLU group reflects greater DA release in the NA and elevated motivation in the GLU group, which might increase lickings in the test session in the GLU group compared to the H_2_O group.

## Introduction

Eating behaviors are occasionally characterized by a trend of elevated food consumption. Such behavior leads to the prevalence of eating disorders such as obesity and binge eating disorder (BED). Recent studies suggest that BED shows specific features involving uncontrolled compulsive food consumption and feelings of loss of control over eating behavior (American Psychiatric Association, 2013), underlined by neurobiological features of dysfunctional cognitive control, food addiction, and gene–environment interactions posing as risk factors (Latagliata et al., 2010; Patrono et al., 2015, 2016; Duarte et al., 2014, 2015). Studies have shown that a low availability of dopamine D2 receptors (DA D2Rs) in the nucleus accumbens (NA) is a genetic risk factor for chocolate compulsive-seeking behavior, which is also mediated by stressful environments (Hoebel et al., 2009; Campbell et al., 2010; Kenny, 2011; Di Segni et al., 2014). This supports the idea that a complex gene–environment interaction plays a key role in the development of maladaptive compulsive eating behavior (Campbell et al., 2010; Patrono et al., 2015).

Food exerts its reinforcing effects on the brain reward system via both gustatory (oral-sensory) and post-ingestive pathways (Li et al., 2002; Jang et al., 2007; Margolskee et al., 2007; Dotson et al., 2010; Fernstrom et al., 2012). A previous neurophysiological study reported that intragastric (IG) infusion of amino acids changed neuronal activity in the lateral hypothalamus and amygdala (Davaasuren et al., 2015). Furthermore, studies have suggested that hepato-portal glucose sensors, which act as an unconditioned stimulus for the acquisition of a learned-food-preference (Delaere et al., 2013) and regulates several physiological functions such as glucose utilization (Burcelin et al., 2000), may directly influence dopaminergic activity. A functional magnetic resonance imaging (fMRI) study using rats reported that IG infusion of glucose activated the NA in less than 10 min (Tsurugizawa and Uneyama, 2014). This suggests a potential role for autonomic afferents innervating the hepato-portal system, in peripheral-glucose-sensing and communication with brain-reward-circuits (Delaere et al., 2013). However, recent evidences have demonstrated an important role for afferent information from the gut in mediating DA release in the reward-system, which stimulates food intake without depending on oral sensation (de Araujo et al., 2008; Ren et al., 2010; Sclafani et al., 2011; Tellez et al., 2013). Thus, nutrient-related DA efflux is induced directly by gastrointestinal tract stimulation, suggesting a gut-brain-DA-axis involved in feeding behavior (de Araujo et al., 2012).

Behavioral studies have reported that positive signals (e.g., glucose intake) serving as unconditioned stimuli (US) in flavor-preference learning tasks are generated in the intestine, and that post-absorptive glucose could condition food and place preferences in rats (Ackroff et al., 2010; Oliveira-Maia et al., 2011; Zukerman et al., 2013). Specifically, the conditioning procedure increased the intake of the flavored conditioned stimulus. In this procedure, oral-intake of flavored conditioned stimuli (CS) is accompanied by IG self-infusion of glucose (US), which suggests that the rewarding effects of glucose are mediated by post-oral processes (Ackroff and Sclafani, 2014, 2015; Sclafani and Ackroff, 2016). These findings suggest that post-ingestive nutrient-conditioned preference and subsequent intake-stimulation are mediated by an “appetition” system, which is different from an intake-suppressor “satiation” system (Sclafani, 2013). Recently, a new conditioning paradigm has been introduced to investigate the post-ingestive nutrient-control-of-food reward (de Araujo et al., 2008; Sclafani et al., 2015). In this paradigm, subjects are trained to lick a sipper spout to receive IG nutrient infusions. During the test sessions, the spout is empty (i.e., without any solution), which allows a direct probe of sensitivity to post-oral detection of infused nutrients. The operant dry-licking paradigm exploits spout licking (CS)-IG self-infusion of a nutrient (US) combination without feedback from oral sensation.

The aim of the present study was to investigate whether IG self-administration of glucose in deprived rats, through dry licking, affected NA neuronal firings in association with dopamine release. Thus, we recorded NA neuronal activity while animals exhibited the operant dry-licking behavioral paradigm. The role of the dopaminergic mesolimbic system in oral and post-oral nutrient conditioning has been extensively investigated (de Araujo et al., 2008; Ren et al., 2010; Sclafani et al., 2011; Tellez et al., 2013, 2016). However, to our knowledge, no previous studies have investigated single-unit neural activity in NA during IG self-administration using the operant dry-licking paradigm.

## Materials and Methods

### Animals

Adult, male Wistar rats (*n* = 14, 250–350 g, SLC, Japan) were used for this study, and equally divided into two groups: glucose group (GLU, *n* = 7) and water group (H_2_O, *n* = 7). GLU received IG self-infusion of glucose, while H_2_O received IG self-infusion of tap water. Housing temperature was maintained at 23 ± 1°C, with a 12-h light/dark cycle (lights on at 07:00). Prior to surgery, two male rats were housed per cage. After the surgery, rats were individually housed, with food and water available ad libitum. All rats were treated in strict compliance with the United States Public Health Service Policy on Human Care and Use of Laboratory Animals, National Institutes of Health Guide for the Care and Use of Laboratory Animals, and the Guidelines for the Care and Use of Laboratory Animals at the University of Toyama. All experimental procedures were approved by our institutional committee for experimental animal ethics. Every attempt was made to minimize the number of experimental animals and their suffering.

### Surgery

Surgical procedures have been described previously (Matsumoto et al., 2012; Davaasuren et al., 2015). Briefly, rats were anesthetized with sodium pentobarbital (40 mg/kg; intraperitoneal, i.p.). Electrode assemblies were implanted bilaterally into NA core (AP = +1.5, ML = ±1.3, DV = +6.5), according to the atlas of Paxinos and Watson (2006). The recording electrode assembly comprised four tetrodes, each of which included four tungsten microwires (20 μm in diameter; California Fine Wire), encased in a stainless steel cannula (30 gauge; Hakko, Japan), and a microdrive. The tip impedance was approximately 200 kΩ at 1 kHz. For intra-gastric cannulation, a midline incision was made in the abdominal wall. One end of a silicon tube was inserted into the gastric fundus and ligated with a silk thread. The other end of the silicon tube was passed from the abdomen under the back skin and held on the skull (Tsurugizawa et al., 2008). After the surgery, all rats were allowed to recover for 1 week and administered intra-muscular antibiotic (orbifloxacin 5%, 0.3 mg/kg; DS Pharma Animal Health, Japan). During the recovery period, the animals were monitored for signs of pain, distress, or morbidity every 12 h. When any of these signs were detected, the animals were immediately sacrificed with an i.p. overdose of sodium pentobarbital.

### Apparatus

**FIGURE 1:**
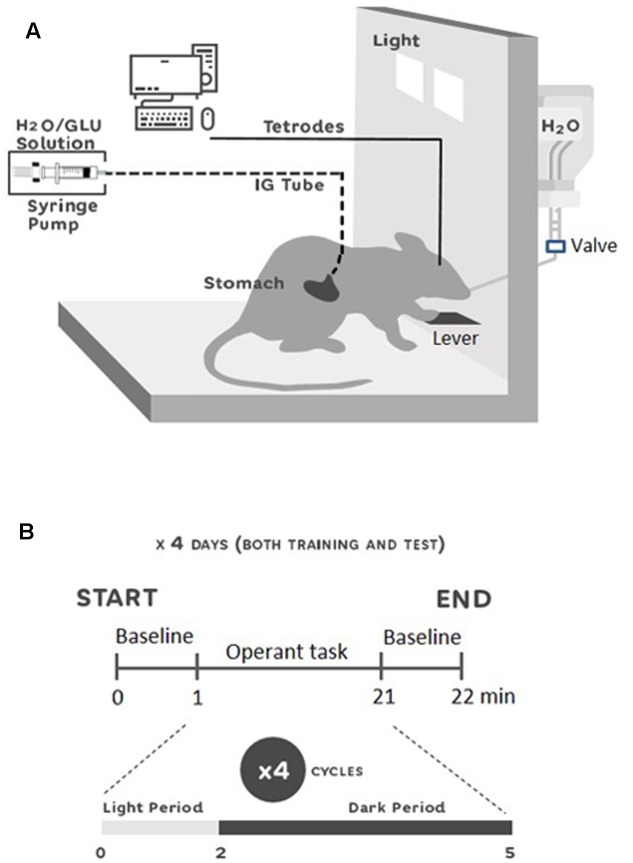
Experimental setup **(A)** and single unit recording schedule **(B)**. **(A)** Skinner box used for the experiments equipped with a flat plastic floor, a stainless steel lever, a sipper spout protruding into the cage, and two halogen lights. IG-solutions were delivered through a gastric cannula using a syringe pump. Neural activity was recorded through a cable connected to the rat’s head. The rat could freely move in the chamber. **(B)** Recording schedule during the 4 days of training and test period. At 0 min the recordings were started with 1 min for baseline recording. Then, the protocol for the operant task in four cycles of 5 min started. At 21 min, the last baseline activity was recorded for 1 min. Each cycle was comprised of 2 min of light on (Light-Period), and 3 min of light off (Dark-Period). In the Light-Period, the animals of both groups had access to a spout with oral-intake of water and IG infusions in the training session, and access to a spout only with IG infusions in the test session. In the Dark-Period, animals rested.

A skinner box (30 cm × 25 cm × 35 cm) was used for behavioral testing (**Figure 1A**): left/right side walls were made of stainless steel, and front/back walls were made of transparent plexiglass allowing video tracking of the animals. The right wall was equipped with two halogen lights (5 watt) and a sipper spout that protruded into the cage by pressing a lever placed just below. On the external side of the right wall, a 20 mL syringe with tap water was connected to the sipper spout. Outside the cage, an automated control-system was set up to control the operant task (turning lights on/off, spout protrusion, spout-licking detection, and delivery of solutions through the spout and IG catheter). Licks on the spout were detected by a touch sensor connected to the spout, and were counted by a computer. Moreover, an injection pump with a 20 mL syringe for IG-infusions was connected to the control-system. The automated control-system was connected to a computer (Interface GPC-2000, Interface, Japan) and ran through a Visual C++ configuration (Microsoft, Corp., United States).

### Behavioral Procedures

All the behavioral procedures were conducted in a sound- and light-attenuated experimental room, during the light phase (4:00–7:00 pm). Following the surgery, physiological saline (1 mL) was flushed through the IG cannula to clean its insides, 1–3 h before behavioral procedures on all experimental days. After 1 week of post-surgery recovery, the animals were trained for several days to form an operant lights-on lever-pressing and sipper-spout association (habituation period). During the habituation period, rats could acquire water (10 μL/lick) from the spout if the rats pressed the lever when the light was turned on. After 1 day of the post-habituation period, the animals received several incidental IG-infusions of 0.4 M glucose solution (5 mL, 1 mL/min/kg) in a glucose novelty test (GNT), to avoid any novelty effect due to IG glucose infusion. After 2 days of the post-GNT period, behavioral testing began. It consisted of training (4 days) and test (4 days) sessions separated by a rest day. Before the training session, the animals were randomly divided into two groups (GLU and H_2_O). Each training day consisted of one session of 20 min. Each session was comprised of four cycles of an operant task. Each cycle included two periods: (1) light and (2) dark periods. In the light period, light was turned on for 2 min, and the animals were allowed to press the lever in order to introduce the sipper spout into the apparatus, to freely lick water, and simultaneously receive IG-infusions. Each lick simultaneously delivered 10 μL of water from the spout and 10 μL of IG-infusion. The solution for IG-infusion was 0.4 M glucose for the GLU group, and tap water for the H_2_O group. In the dark period, light was turned off for 3 min and the animals rested. Since it has been reported that IG infusion of glucose increased blood-oxygen-level dependent (BOLD) signals in NA in less than 10 min (Tsurugizawa and Uneyama, 2014), and because BOLD signals have a poor temporal resolution, we considered that a 5-min-cycle could be a useful time window for IG glucose to affect neuronal firings in NA. In the test session, the same protocols as those in the training session were used, except that the sipper spout was dry (i.e., without water). The animals were water-deprived for 20 h during all the procedures, except for the rest days during which they returned to drink water ad libitum. **Figure 1B** shows the timelines of the behavioral procedures.

### Recording Procedure

A cable was connected to the socket on the rat’s head, which was connected to the electrodes on the subject rat’s head, and neuronal activity was recorded. The analog signal was sent to a set of amplifiers (Omniplex, Plexon, United States) and then to an analog-to-digital system (Omniplex, Plexon, United States). Neuronal activities were digitized at a 40-kHz sampling-rate. Any 0.8-ms waveforms that crossed an experimenter-defined threshold were stored for offline spike sorting via OmniPlex (Plexon, Inc., Dallas, TX, United States). Moreover, a digital camera was connected to the system, in order to track animal behavior. If no signal was found, the electrode assembly was lowered by approximately 80–100 μm, and checked again on the following day. If stable neuronal signals were identified over a 10-min period, the electrode assembly was fixed.

Neuronal activity was recorded only in training and test sessions. At the beginning of the session, animals were connected to the cable and placed into the apparatus. After a 10-min period for stabilizing the neuronal signals, the session started with 1 min baseline, a 20 min session (four cycles of the operant task), and a 1 min baseline (**Figure 1B**). When the session was completed, the rat was returned to its own cage. Neuronal activity was recorded from the same electrode location, throughout the training and test sessions.

### Data Analyses

#### Behavioral Analysis

Behavioral data in the light/dark periods were analyzed in two different ways, across training and test sessions. In the light period, the operant dry-licking behavior was analyzed in both training and test sessions, in order to evaluate if the deprived rat was able to self-infuse IG glucose despite the absence of the oral-intake of water. In the dark period, three behavioral features (exploration, grooming, and immobility) were analyzed in the test session, to assess physical behavioral activity.

In the light period, to test the hypothesis that deprived rats are able to self-infuse IG 0.4 M glucose despite the absence of the oral-intake of rewarding solution (water), two behavioral parameters were measured: (1) total number of licks/IG-infusions in the GLU and H_2_O groups in the training and test sessions; (2) lick-tendency across the 4 days in training and test sessions in GLU and H_2_O groups. Total numbers of licks/IG-infusions were compared by repeated measures two-way (RMT-) ANOVA with two factors: group (GLU vs. H_2_O) and task condition (training vs. test). In lick tendency, averaged daily licks in the training or test session were compared by RMT-ANOVA with two factors: group (GLU vs. H_2_O) and day (days 1–4).

In the dark period, to check a possibility of non-specific correlation between NA neuronal activity and physical behavioral activity, three main behavioral features (exploration, grooming, immobility) were compared between GLU and H_2_O groups in the test session. Exploratory behavior included any locomotor activity around the chamber; including rearing, sniffing, and approaching the sipper spout hole location. Grooming behavior was defined as any self-care behavior, and immobility was defined as any resting behavior. The duration (%) and frequency (per min) of the three behaviors were measured using Observer 5.0 (Noldus, The Netherlands). Behavioral data in the two groups were compared using the unpaired *t*-test, since the three behaviors are not independent.

Statistical analysis was conducted with MatLab R2015b (Mathworks, Inc., United States), or Microsoft Excel 2010 (Microsoft, Corp., United States). *p*-values less than 0.05 were considered statistically significant.

#### Neurophysiological Analyses

The recorded waveforms were projected to a principle component subspace using NDManager (Hazan et al., 2006^1^) and semi-automatically sorted into single neurons using KlustaKwik (Harris et al., 2000^2^), and Kluster (Hazan et al., 2006^1^) respectively, according to previous studies (e.g., Maingret et al., 2016).

It is reported that distinct types of striatal neurons differently respond to reward (Berke, 2008; Lansink et al., 2010; Matsumoto et al., 2012), and show different spontaneous firing patterns (Berke et al., 2004; Berke, 2008; Schmitzer-Torbert and Redish, 2008; Gage et al., 2010; Lansink et al., 2010; Matsumoto et al., 2012). To separately analyze the different types of neurons, they were classified based on the following three electrophysiological properties according to previous studies (Berke, 2008; Schmitzer-Torbert and Redish, 2008; Gage et al., 2010; Matsumoto et al., 2012): (1) post-spike suppression (Schmitzer-Torbert and Redish, 2008), the period that passed before neuronal activity returned to its average firing rate after each action potential; (2) spike width (peak-to-valley duration of the waveform; Gage et al., 2010); (3) mean firing rate during baseline phase. According to the previous reports (see above), putative medial spiny neurons (pMSNs) were defined as such if the spike widths were >0.37 ms, and the post-spike suppression was <50 ms. Putative fast spiking interneurons (pFSIs) were defined as such if the mean firing rates were >2 Hz, the spike widths were <0.33 ms, and the post-spike suppression was <50 ms. The neurons that did not match any of the criteria above were defined as unclassified neurons.

A previous study reported that IG-infusion induced changes in motivation and/or post-ingestive effects (Tsurugizawa and Uneyama, 2014). We hypothesized that changes in motivation and/or post-ingestive effects might be mediated by firing patterns of NA neurons in the dark period. To analyze firing patterns of NA neurons, mean firing rates and coefficient of variance (CV) (SD/mean) of inter-spike intervals in the dark period (Stern et al., 1997) were calculated and compared between the two groups using two-way ANOVA with two factors: two groups (GLU vs. H_2_O) × 2 task conditions (training vs. test).

## Results

### Operant Dry-Licking and Lick-Tendency

**FIGURE 2:**
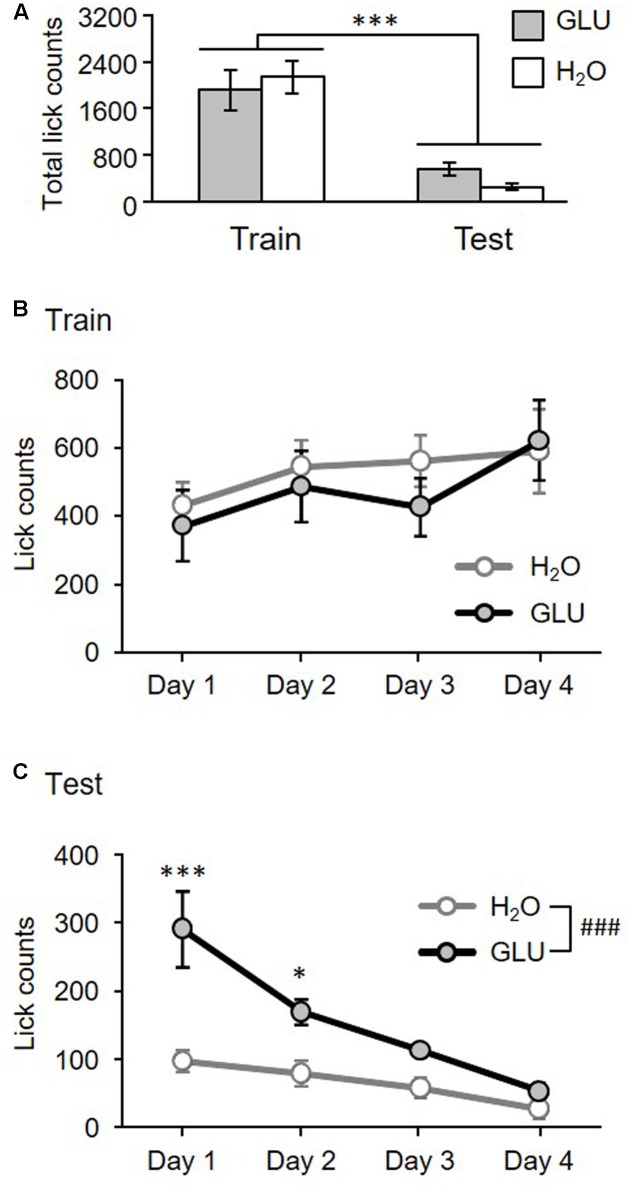
Total number of licks for 4 days **(A)** and lick-tendency across 4 days **(B,C)** in the training and test sessions for GLU and H_2_O groups. **(A)** Total number of licks for 4 days. The histograms show the total lick counts for 4 days in H_2_O (white bars) and GLU group (gray bars), in Training (Train) and Test (Test) sessions. There was a significant main effect of task condition (Training vs. Test). ^∗∗∗^*p* = 4.5 × 10^-6^. **(B,C)** Lick-tendency in training **(B)** and test **(C)**. Ordinates indicate averaged daily in each experimental day. **(B)** No significant differences have been found. **(C)** There were significant main effects of group, and day, and significant interaction between group and day. ###*p* = 0.0017; ^∗∗∗^*p* = 8.0 × 10^-7^; ^∗^*p* = 0.011. Error bars represent ± SEM.

**Figure 2A** shows the total number of licks/IG-infusions in the GLU and H_2_O groups during the training and test sessions. A statistical comparison by RMT-ANOVA indicated a significant main effect of task condition (training vs. test) (*F*[1,12] = 61.70, *p* = 4.5 × 10^-6^). There was no significant main effect of group (GLU vs. H_2_O) (*F*[1,12] = 0.09, *p* = 0.76), or an interaction between the group and task condition (*F*[1,12] = 2.09, *p* = 0.17).

**Figure 2B** shows lick-tendency across 4 days in the training session. A statistical comparison by RMT-ANOVA indicated that there was no significant main effect of group (*F*[1,12] = 0.26, *p* = 0.62) nor interaction between group and day (*F*[3,36] = 0.49, *p* = 0.69). The results indicated that there was no significant difference between GLU and H_2_O groups during the training session. However, there was a significant main effect of day (*F*[3,36] = 2.98, *p* = 0.044) indicating that lick-counts gradually increased throughout training.

**Figure 2C** shows lick tendency across 4 days in the test session. A statistical comparison by RMT-ANOVA indicated that there were significant main effects of group (*F*[1,12] = 16.11, *p* = 0.0017) and day (*F*[3,36] = 6.17, *p* = 0.0017), and a significant interaction between group and day (*F*[3,36] = 19.62, *p* = 1.0 × 10^-7^). The *post hoc* test comparisons revealed that lick-counts in days 1 and 2 were significantly larger in the GLU group than the H_2_O group (day 1, *p* = 8.0 × 10^-7^; day 2, *p* = 0.011; simple main effect test). The results indicate that overall, the GLU group licked the spout more without oral intake than the H_2_O group, although licking gradually decreased across 4 days in both groups.

### Behaviors in the Dark Period

**FIGURE 3:**
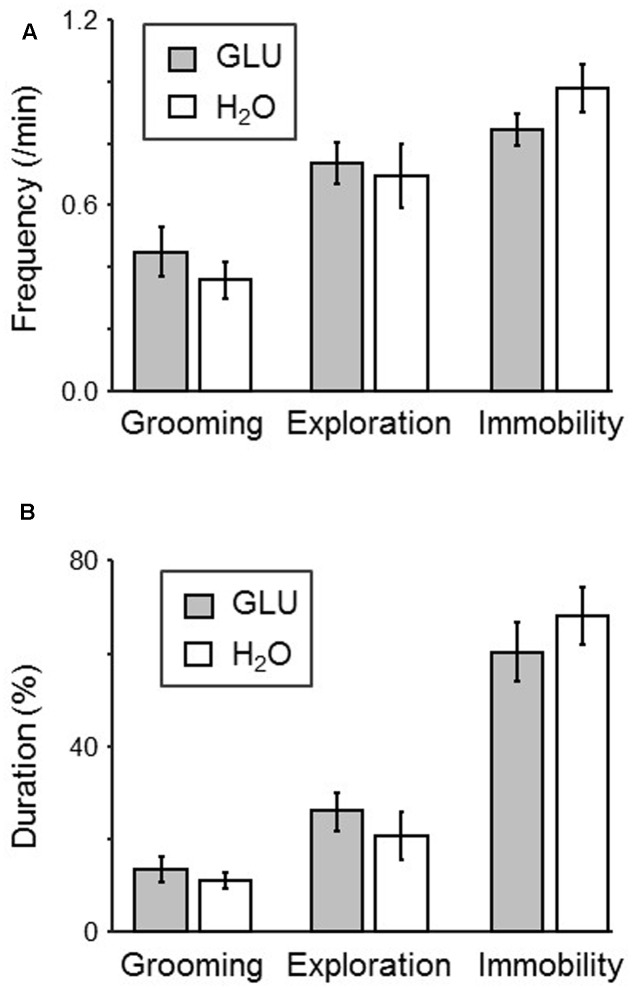
Comparison of behaviors in the dark-period of the test session between H_2_O and GLU groups. **(A,B)** Frequency **(A)** and duration **(B)** of each behavior (grooming, exploration, and immobility). No significant difference between the groups was found.

**Figure 3** shows comparisons of each behavior (exploration, grooming, and immobility) in the dark period of the test session between groups. The results indicated that there was no significant difference in the frequency of each behavior between groups (*p* > 0.05, unpaired *t*-test) (**Figure 3A**). Furthermore, the results also indicated that there was no significant difference in the duration of each behavior between groups (*p* > 0.05, unpaired *t*-test) (**Figure 3B**).

### Neuronal Firing Patterns

**FIGURE 4:**
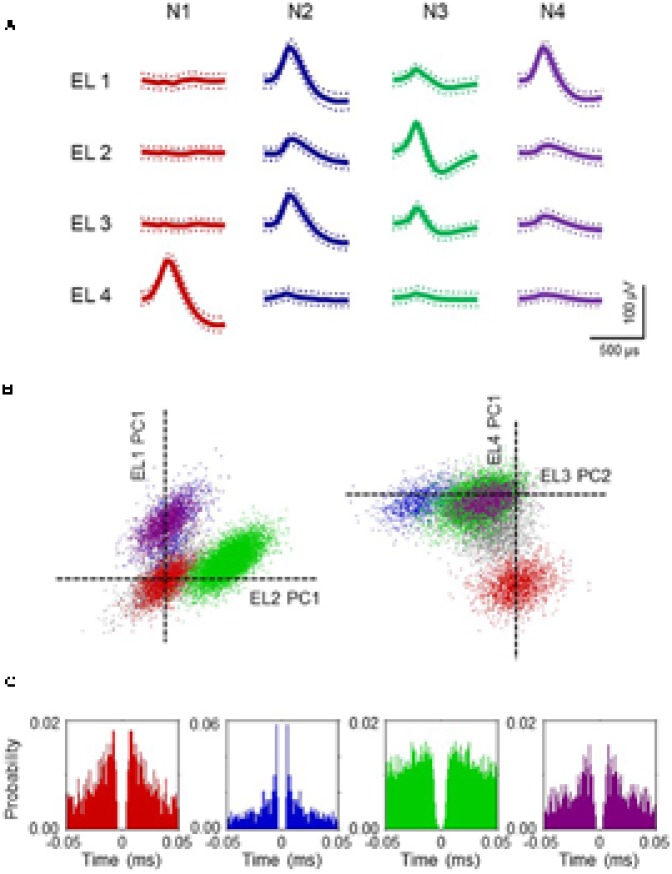
Example waveforms of four NA neurons isolated by an offline cluster analysis. **(A)** Waveforms (mean ± SD, dotted) recorded from four electrodes (tetrode) (EL 1–4). The waveforms indicated by N 1–4 correspond to four neurons, identified by the offline cluster analysis in **(B)**. **(B)** The results of an offline cluster analysis. Each axis represents a principle component (PC1 or 2). Each dot represents one neuronal spike. The semi-automatic spike sorting resulted in the four colored clusters of neural signals. Gray dots represent unsorted spikes. The axes represent the first principle component of each of the four electrodes. Four colored clusters (red, blue, green and purple, corresponding units N 1–4 in **A**, respectively) are recognized. **(C)** Autocorrelograms of the neurons. Bin width = 1 ms. Ordinates indicate probability, where bin counts were divided by the number of spikes in the spike train.

**FIGURE 5:**
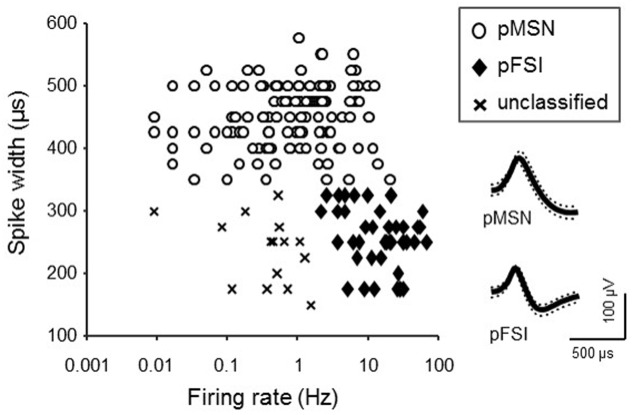
Classification of NA neurons. The graph indicates a scatter plot showing electrophysiological properties (spike width and baseline firing rate) of each NA neuron. Symbols represent classes of NA neurons. Inset shows representative waveforms (mean ± SD, dotted lines) of pMSN and pFSI.

The firing patterns of 191 neurons were recorded from NA. Typical waveforms of four NA neurons (N 1–4) simultaneously recorded from four wires (EL 1–4) in the same tetrode are shown in **Figure 4A**. **Figure 4B** displays the results of spike-sorting by offline cluster cutting of the neuronal activities shown in **Figure 4A**. Each dot represents one spike, and four clusters of dots indicated by different colors were recognized. Autocorrelograms of these neurons indicated that their refractory periods were more than 3 ms, which demonstrates that these spikes were recorded from single neurons (**Figure 4C**). **Figure 5** shows three types of NA neurons based on neurophysiological parameters. A scatter plot of NA neurons, based on the neurophysiological criteria of mean firing rates and spike widths, indicated that clusters of pMSNs (open circles) and pFSIs (closed diamonds) were clearly identified. **Table 1** shows numbers of each type of NA neurons recorded in each period, per group. In the following analyses, we focused on pMSNs, because the numbers of neurons recorded in other classes were not enough to compare neural activity between groups.

**Table 1:** Number of neurons recorded in this study.

	Glucose group		H_2_O group		
	Training	Test	Training	Test	Total
pMSN	27	23	52	20	122
pFSI	14	4	12	12	42
Unclassified	9	1	10	7	27
Total	50	28	74	39	191


*pMSN, putative medial spiny neurons; pFSI, putative fast spiking interneurons*.

**FIGURE 6:**
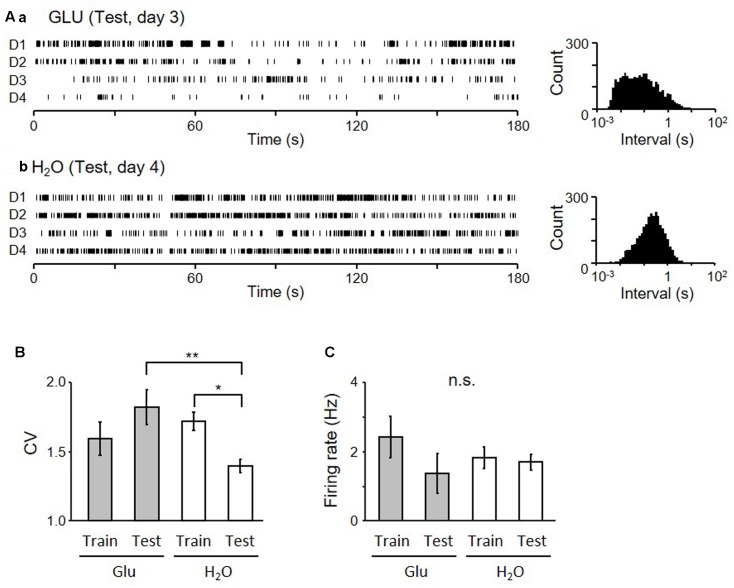
Firing patterns of pMSNs in the dark period of the Test session. **(A)** Raster plots of neuronal activity of two neurons (a from GLU-group and b from H_2_O-group) in the dark-period of four cycles (D1–4) (left) and inter-spike interval histograms of the given neurons (right). **(B)** Comparison of coefficient of variance (CV) of the inter-spike intervals of all neurons recorded during the 4 days of each session in each group. ^∗^*p* < 0.05, ^∗∗^*p* < 0.01. **(C)** Comparison of mean firing rates.

Examples of firing patterns of two pMSNs in the dark period are shown in **Figure 6**. The inter-spike intervals of a neuron shown in **Figure 6A** recorded from the GLU-group are more variable than those of a neuron shown in **Figure 6B** recorded from the H_2_O group, during the Test. **Figure 6B** shows a statistical comparison of coefficient of variances (CV) of the inter-spike intervals by two-way-ANOVA. The results indicated that there was a significant interaction between group (GLU vs. H_2_O) and task condition (training vs. test) (*F*[1,118] = 8.05, *p* = 0.0053), although there was no significant main effect of group (*F*[1,118] = 2.39, *p* = 0.12) and task condition (*F*[1,118] = 0.23, *p* = 0.63). *Post hoc* analysis revealed that CV during test session in the H_2_O group was significantly smaller than the CV during the training session in the same group (*p* = 0.020, simple main effect test) and the CV during test in the GLU group (*p* = 0.0024, simple main effect test). However, there was no significant difference in CV between training and test sessions in the GLU group (*p* = 0.098, simple main effect test) nor significant difference in CVs during training session between GLU and H_2_O groups (*p* = 0.36, simple main effect test). **Figure 6C** shows a statistical comparison of mean firing rates by two-way-ANOVA. The results indicated that there was no significant main effect of group (*F*[1,118] = 0.08, *p* = 0.78) and task condition (*F*[1,118] = 1.57, *p* = 0.21), or significant interaction between the group and task condition (*F*[1,118] = 0.95, *p* = 0.33). We also tested whether CV and/or mean firing rates changed across the four experimental days in the test session by two-way-ANOVAs with two factors: group (GLU vs. H_2_O) and day (days 1–4). The statistical results indicated that there were no significant differences in CV and mean firing rates among the 4 days (i.e., no significant main effect of day, nor no significant interaction between day and group: data not shown). These results indicate that the firing variability of pMSNs in the dark period was maintained in the test session without oral water intake in the GLU group, while the firing variability of pMSNs was reduced in the test session in the H_2_O group.

**FIGURE 7:**
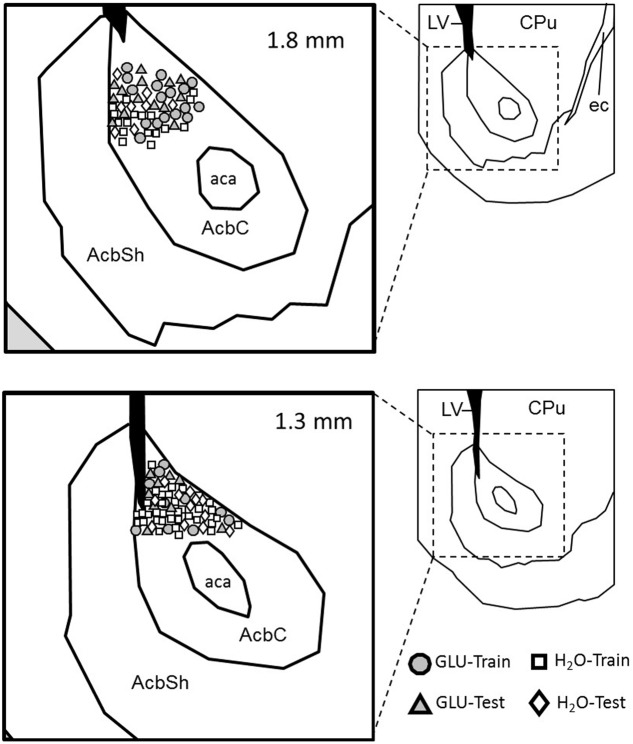
Locations of recorded pMSNs. Estimated positions of the recorded pMSNs in each condition (Training or Test) in each group (GLU and H_2_O) in coronal sections. Each inset in the right shows a low-magnified view of the corresponding section. The value on each section indicates the distance from the bregma. AcbC, nucleus accumbens core; AcbS, nucleus accumbens shell; aca, anterior commissure anterior part; CPu, caudate putamen; ec, external capsule.

The electrode locations in each recording session were calculated based on the implanted coordinate and the total microdrive advancement (**Figure 7**), indicating that all NA neurons were recorded from the core of NA.

## Discussion

In the present study, we test the hypothesis that a dry-licking behavior associated with IG glucose affects NA neuronal firing using a new operant dry-licking paradigm where IG self-administration of glucose was able to induce a licking behavior, in spite of the absence of oral-intake. The present results indicate that IG glucose self-administration induced different firing pattern of NA neurons than water IG glucose self-administration.

### Operant Dry Licking Behavior

In this study, a new paradigm of dry-licking behavior was used to assess the ability of IG self-administration of 0.4 M glucose to enhance licking behavior in spite of the absence of oral-intake. Daily dry-licks across 4 days in the test session were increased in the GLU group with IG self-administration of glucose, compared to the H_2_O group with IG self-administration of water. Consistent with the present results, previous studies report similar results that IG nutrient without oral feedback exerts rewarding effects (Tellez et al., 2013). The present results extend previous findings; rewarding effects of IG infusion of glucose support not only licking a dry-spout but also instrumental conditioning (i.e., lever pressing upon light on).

Rewarding effects of IG infusion of glucose might be mediated by DA release in NA. Previous studies reported that IG infusion of glucose or sucrose induced DA release in NA (Ren et al., 2010). It is believed that food and water, or cues associated with them, activate DA neurons, and facilitate behaviors directed toward the acquisition of reward (Palmiter, 2007). Thus, DA might affect NA neuronal firings, which might modulate licking behaviors (Frazier and Mrejeru, 2010).

### Neuronal Firing Patterns in NA

We found that CV of pMSN activity decreased in the dark period of the test session without oral intake of water, compared to that in the training session of the H_2_O group, while the CV was maintained in the same test session in the GLU group. Licking-behavior also showed similar changes; licking-counts in the light-period were decreased in the test session of the H_2_O group compared to the GLU group, suggesting that the firing variability (i.e., CV) in the dark-period reflects motivational state and/or post-ingestive effect (see below in details).

The reduction of the licking behavior in the light period of the test session of the H_2_O group may be associated with a reduction in DA release in NA during the dark period. Previous studies suggest a relationship between DA release and appetitive behaviors; tonic (slow) increases in DA level are involved in motivation (Niv et al., 2007; Beierholm et al., 2013; Hamid et al., 2016), seeking for reward is positively correlated with the DA level (Hamid et al., 2016), and DA modulates vigor (Beierholm et al., 2013; Hamid et al., 2016). A previous study also reported increases in DA level during delay before starting the task (Hamid et al., 2016). The present behavioral results indicated that animals performed the task less vigorously in the test session without oral intake in the H_2_O group. Taken together, these results suggest that DA release might be reduced in the dark period of the test session in the H_2_O group.

In the present study, firing variability (CV) of pMSNs in the dark period of the test session was decreased in the H_2_O group. Previous studies suggest that DA may increase CV of activity of medial spiny neurons (MSNs) in the NA. Firing variability is associated with transition between the up-state and the down-state of MSNs (Stern et al., 1997) and the transition may be potentiated by DA (Tritsch and Sabatini, 2012). Finally, CV is defined as a good parameter in the quantal analysis of excitatory post-synaptic potentials (EPSPs) in the striatal DA-ergic transmission (Murer et al., 2002). These findings suggest that DA release increases firing variability by increasing up- and down-state transition. Furthermore, such an elevated DA level itself is involved in spontaneous fluctuation of DA release (Stuber et al., 2005), and MSN activity in NA is modulated by DA transmission (Tritsch and Sabatini, 2012), suggesting that such a DA fluctuation might also account for firing variability. Furthermore, physical behaviors were similar between GLU and H_2_O groups in the dark-period of the test session, suggesting that the difference in firing variability of pMSNs was not ascribed to differences in physical behaviors between GLU and H_2_O groups.

In the present study, there was no significant difference in CV across the 4 days in the test session although there was a significant main effect of group (GLU vs. H_2_O groups). This suggests that the rats in the GLU group might continue to be motivated to acquire IG glucose infusion as well as water from the spout across the 4 days in the test session although lick counts were decreased in day 4. Further studies with longer experimental days might clarify changes in CV across the experimental days in the test session.

Taken together, these results suggest that decreases in firing variability of pMSNs in the H_2_O group may reflect decreases in DA release in H_2_O group, which consequently may decrease lick counts in test session in H_2_O group. In conclusion, the present results have demonstrated that IG glucose self-administration is able to induce neuronal activation in the brain rewarding system, suggesting the role of a “gut-brain DA axis” in appetitive behaviors. However, further studies such as simultaneous recording of DA release and MSN firing is required to confirm this hypothesis.

## Author Contributions

EP conceived the study. EP, JM, and HisN designed the experiment. EP performed the experiment. EP and JM analyzed data and wrote the paper. HisN, HirN, YT, and TO revised the paper. All the authors discussed the results and commented on the manuscript, and read and approved the final manuscript.

## Conflict of Interest Statement

The authors declare that the research was conducted in the absence of any commercial or financial relationships that could be construed as a potential conflict of interest.

## Footnotes

1 http://neurosuite.sourceforge.net/

2 http://klustakwik.sourceforge.net/
